# Diversity of Fungi Isolated from Potato Nematode Cysts in Guizhou Province, China

**DOI:** 10.3390/jof9020247

**Published:** 2023-02-13

**Authors:** Hui Zhang, Zaifu Yang, Zhaochun Jiang, Xinyue Zhang, Mir Muhammad Nizamani, Yan Wu, Shan Wei, Yong Wang, Xin Xie

**Affiliations:** 1 Department of Plant Pathology, College of Agriculture, Guizhou University, Guiyang 550025, China; 2 Vegetable Research Academy, Guizhou University, Guiyang, 550025, China; 3 Guizhou Station of Plant Protection and Quarantine, Guiyang, 550001, China

**Keywords:** nematode–fungi interactions, community composition, functional annotations, parasitic potential

## Abstract

Potatoes rank third in terms of human consumption after rice and wheat. *Globodera* spp. are significant pests of potato crop worldwide. *Globodera rostochiensis* was found in Weining County, Guizhou Province, China, in 2019. We collected soil from the rhizosphere zone from infected potato plants and separated mature cysts through simple floatation and sieving methods. The selected cysts were surface-sterilized, and the colonized fungi were isolated and purified. At the same time, the preliminary identification of fungi and fungi parasites on the cysts of nematodes was carried out. This study aimed to define the species and frequency of fungi-colonizing cysts of *G. rostochiensis* collected from Weining County, Guizhou Province, China, and provide a basis for the control of *G. rostochiensis*. As a result, 139 strains of colonized fungi were successfully isolated. Multigene analyses showed that these isolates included 11 orders, 17 families, and 23 genera. The genera *Fusarium* (with a separation frequency of 59%), *Penicillium* (11%), *Edenia* (3.6%), and *Paraphaeosphaeria* (3.6%) were the most frequently occurring. Among the 44 strains, 27 had a colonization rate of 100% on the cysts of *G. rostochiensis*. Meanwhile, the functional annotation of 23 genera indicated that some fungi have multitrophic lifestyles combining endophytic, pathogenic, and saprophytic behavior. In conclusion, this study showed the species composition and lifestyle diversity of colonized fungi from *G. rostochiensis* and demonstrated these isolates as potential sources of biocontrol agents. Colonized fungi were isolated from *G. rostochiensis* for the first time in China, and the taxonomic diversity of fungi from *G. rostochiensis* was clarified.

## 1. Introduction

The potato is one of the most widely grown staple foods [1]. Potatoes provide more calories, protein, and minerals than any other staple crop [2]. As populations grow and urbanization intensifies, potato production has surged due to increased global consumption [3]. However, production is still adversely affected by pests and pathogens, including the potato cyst nematodes (PCNs) *Globodera rostochiensis* and *G. pallida* [4]. Both species contain pathotypes, and some closely related and very similar species are of minor economic importance.

PCNs—*Globodera* spp.—are among the most significant pests of potato crops worldwide [5]. PCN species are believed to have evolved in South America but now have a worldwide distribution and can be major and persistent pests except in the warmest soils [6]. The life cycle of PCNs is well-adapted to the host, and they can survive in various environments. Root exudates from Solanaceae activate juveniles, which can cause up to 80% of the nematodes to hatch under suitable environmental conditions [7]. Yield loss due to PCNs has been reported as 90–100% in Europe and North America [8]. In India, up to 80% yield loss due to PCNs was reported from the Nilgiris and Kodaikanal Hills, the Tamil Nadu region [9], Karnataka, Kerala, and Himachal Pradesh [10,11,12]. *G. rostochiensis* was also detected in the provinces of Sichuan and Yunnan, China, in 2022 [13]. A survey of cyst nematodes in potato fields was conducted in Guizhou Province from 2018 to 2020; *G. rostochiensis* was first reported in Guizhou Province in 2022 [14].

Nematophagous (nematode-destroying) fungi are natural enemies of nematodes, which have been found in all regions of the world, from the tropics to Antarctica. They have been reported in agricultural, garden, and forest soils and are especially abundant in soils rich in organic material [15]. Biological control of plant-parasitic nematodes (PPNs) using nematophagous fungi has received considerable attention [16] due to the urgent need for alternatives to synthetic nematicides, which are being phased out due to environmental concerns [17]. Since nematophagous fungi were first discovered in soil in 1852 [18]; presently, more than 200 species of fungi have been identified as colonizers of cysts, eggs, and females of eight cyst nematode species in the soil, including PCNs and beet cyst nematodes (BCNs) [19,20]. Many fungi have been isolated from cyst nematodes, and some of them contribute to nematode suppression in the soil [21]; these nematodes are susceptible to fungal parasitism [22,23]. Rajeswari and Sivakumar reported five native nematophagous fungi—*Penicillium* spp., *Aspergillus* spp., *Paecilomyces lilacinus*, *Verticillium suchlasporium* var. *suchlasporium*, and *Exophiala pisciphila*—which were found to parasitize the eggs of *Globodera* spp. [24]. Yu and Josef found fungal species associated with the cysts of *G. rostochiensis* and *G. pallida*: *Alternaria alternata*, *Chaetomium gracile*, *Cylindrocarpon*, *Fusarium*, *Gliocladium roseum*, *Mariannaea elegans*, *Penicillium simplicissium*, *Periconia macrospinosa*, *Phoma medicaginis*, *Trichocladium asperum* and *Verticillium coccosporum* from northern Belgium [16]. Fungal species, such as *Purpeocillium lilacinum*, *Fusarium* spp., and *Hirsutella* spp., were preponderant parasitic fungi in some soils and deemed potential biological control agents [25,26,27]. *Paecilomyces lilacinus* is the most widely tested fungus for the control of root-knot and cyst nematodes. Many authors have investigated its use in the field to control nematode populations [28,29,30], and it has been routinely isolated from infected nematode eggs in soils suppressive to plant-parasitic nematodes [31,32,33,34]. Currently, it is the only commercially available fungal formulation to control nematode pests, and the commercial strain 251 is registered for sale in several countries.

The colonization rate and diversity of *G. rostochiensis*-colonized fungi varies in different environments. In Europe, the difference in the colonization rates of *G. rostochiensis* eggs was found to be significant between northern and southern Sweden, with 17% in the northern part and as low as 3% in the southern part, which was considered the main reason for the rapid 80–90% decline in *G. rostochiensis* eggs in the soil of the northern region. The isolates of *Verticillium sucraleum* from its cysts had a parasitic rate of 93% in its eggs and demonstrated both chitinase and protein enzyme activity [35]. In Britain, Crum et al. planted potatoes in soil from different sources, inoculated the cysts of *G. pallida,* and then isolated and identified the colonized fungi from their females, which verified that the diversity of colonizing fungi was directly related to the fungi species in the soil. At the same time, the biocontrol effect of some strains has been evaluated, and it was found that three strains effectively reduced the population of the above two nematodes [36]. It was found that the dominant colonizing fungi and species of *G. rostochiensis* and *G. pallida* varied in different regions of Serbia [37]. In Asia, five colonizing fungi were isolated from the eggs of *G. rostochiensis* and *G. pallida* in India, among which the colonization rate of *Paecilomyces lilacinus* was 79.6% [24].

On the other hand, 34 species of colonizing fungi on the eggs of *G. rostochiensis* were identified in Iran, belonging to 11 different genera. The obtained *F. oxysporum* and *T. atroviridae* strains had high chitinase activity, which could be used to control *G. rostochiensis* [38]. *Trichoderma* spp. and *Fusarium* spp. are the main colonizing fungi of *G. rostochiensis* in Algeria, Africa. The nematicidal activity of the *T. harzianum* and *T. afroharzianum* strains was evaluated through fermentation [39]. A new colonizing fungus (*Volutella citrinella* GUCC2219) was obtained in Guizhou, isolated from *G. rostochiensis* cysts with predatory and nematicidal activities against three plant-parasitic nematodes (*Aphelenchoides besseyi*, *Bursaphelenchus xylophilus*, and *Ditylenchus destructor*) [40]. In summary, due to various geographical locations, the diversity of colonizing fungi in the soil, and planting methods, there are significant differences in colonizing fungi strains and dominant populations in regions where *G. rostochiensis* occurs, and these colonizing fungi often have different degrees of nematicidal activity. The products developed from it have been successfully applied to controlling *G. rostochiensis* [41,42].

In China, fungal antagonists of PCNs have not been investigated so far due to *G. rostochiensis* only being found in 2018. Thus, an effective technique is necessary to suppress the PCN population in Weining and prevent its spread. In addition, interactions between microorganisms and *G. rostochiensis* have not been reported. Weining County has a humid subtropical monsoon climate, with an average of 1812 h of sunshine and 180 frost-free days per year, 926 mm of annual rainfall, small annual temperature differences and large daily temperature differences, and warm winters and cool summers, with an average temperature of 18˚ in the summer [43,44]. The climatic conditions in Weining County are suitable for the occurrence of potato nematodes.

In Guizhou province, crop rotation is difficult to implement in many potato-producing areas; and chemical nematicides have problems of high toxicity, easy residues, and high costs, so biological control is a high priority. According to Mo [45], to solve some of the problems of nematode biological control, the competition for survival among and within groups of organisms in the soil ecosystem needs to be examined from the perspective of biodiversity. The fungal biological control of PCNs is an important component of integrated pest management for potatoes. However, very few fungal biological control agents are available on the market. This study aimed to investigate the strains and frequency of fungi colonizing cysts of *G. rostochiensis* collected from Weining County, Guizhou Province, China, and provide a basis for the control of *G. rostochiensis*.

## 2. Materials and Methods

### 2.1. Nematode Collection

**Figure 1:**
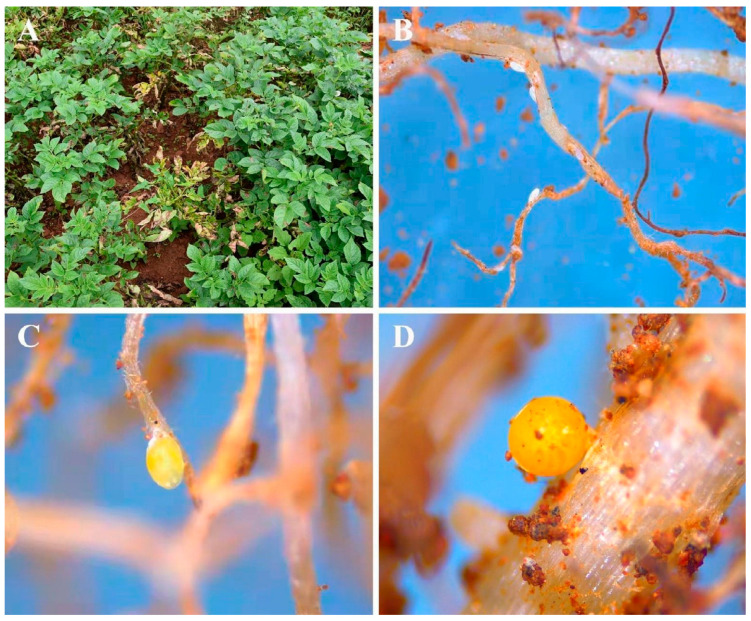
(**A**) Potato infected by *G. rostochiensis*; (**B**–**D**) potato roots infested by *G. rostochiensis*.

The cysts of *G. rostochiensis* were collected from soils of one potato field naturally infected with *G. rostochiensis*, located in Weining County, Guizhou, China (Figure 1). In a field, ten 5 × 5 m grid plots were selected surrounding infected potato plants, and in each grid an approximate volume of 250 mL of soil was collected from the rhizosphere zone (0–20 cm in depth). The individual samples of each plot were collected and mixed in a bucket to obtain a single composite sample [46]. Each composite sample was thoroughly mixed to obtain a homogenous sample. A subsample of approximately 500 mL of soil was then air-dried at 37 °C for two days for PCN cyst extraction [47,48,49]. Cysts were extracted from a subsample of 50 g of dried soil using the Baunacke method [50,51], i.e., dried cysts that floated in water were decanted and collected on a 250 μM sieve.

### 2.2. Isolation of Fungi from Cysts of G. rostochiensis

The selected cysts were surface-sterilized in 2% H_2_O_2_ for 3 min following three washes with distilled water. The surface-sterilized cysts were individually placed onto 1% WA plates. Plates were incubated at room temperature and monitored regularly. Fungal mycelia growing from the cultured cysts were re-cultured several times on new PDA plates. The pure cultures were initially grouped based on their morphological criteria. All fungal isolates were conserved in the Culture Collection of the Department of Plant Pathology, Agriculture College, Guizhou University.

### 2.3. DNA Extraction, PCR, and Sequencing

The fungal isolates were grown on PDA at 25 °C for 7 days. The resulting mycelia were then scraped off the surface of the plate with a sterile scalpel. Total genomic fungal DNA was extracted using a BIOMIGA Fungus Genomic DNA Extraction Kit (GD2416, BIOMIGA, San Diego, CA, USA) following the manufacturer’s protocol. PCRs were conducted in a 25 μL reaction mixture containing 10 μL 2 × Bench Top Taq Master Mix (Biomiga, AT1201, San Diego), 7 μL of ddH_2_O, 1 μL of forward and reverse primers (10 μM/μL), and 1 μL of DNA template. PCR products were commercially sequenced with the same PCR primers used in the amplification reactions by Sangon Biotech Co., Ltd. (Shanghai, China).

### 2.4. Multigene Analyses

Colonizing fungi were identified by protein-coding and ribosomal gene sequences. All forward and reverse sequences were used to create consensus sequences in BioEdit v. 7.0.9.0 [52], and BLASTn searches in NCBI were used to identify the taxonomic status at the genus level.

### 2.5. Diversity Indices and Functional Annotation Analysis

#### 2.5.1. Dominant Taxa

A taxon is defined as dominant if Pi > Camargo’s index (1/S), where S represents species richness, which is the number of fungal taxa, and Pi is calculated as the number of isolates (Ni) that belong to a certain taxon (i) divided by the total number of isolates (N) [53].

#### 2.5.2. Lifestyle Diversity

The lifestyle status of culturable fungi was predicted using the FUNGuild database. The functional annotation of fungi at the genus level was considered appropriate [54].

### 2.6. In Vitro Parasitic Potential Tests of the Fungal Isolates towards Nematode Cysts

Fungi with different morphological characteristics were selected to screen potential parasitic fungi of *G. rostochiensis* cysts. The purified strains were cultured on a PDA medium. When the colony diameter grew to 1/2–3/4 of the culture dish, the surface-sterilized (2% H_2_O_2_ for 3 min) cysts were individually placed onto the edge of the fresh hypha on the PDA medium, and 10 cysts were placed per dish, with three replicates per strain. After the cysts and plates were incubated at 25 °C for 10 days, the cysts were gently picked out (ensuring not to break them) and surface-sterilized in 0.5% NaClO for 3 min following three washes with distilled water. Each cyst was individually placed onto sterilized filter paper. After moisturizing the culture for five days, the cysts’ colonization rate was recorded by observing the fungi growth on the cysts on the filter paper.

### 2.7. Data Analysis

All the statistical analyses were conducted in MS Excel and SPSS Statistics (version 19.0) software. Figures were generated using MS Excel, Adobe Photoshop 2021 and Chiplot Web (https://www.chiplot.online/, accessed on 26 December 2022).

## 3. Results

### 3.1. Fungi Associated with Cysts of G. rostochiensis

**Figure 2:**
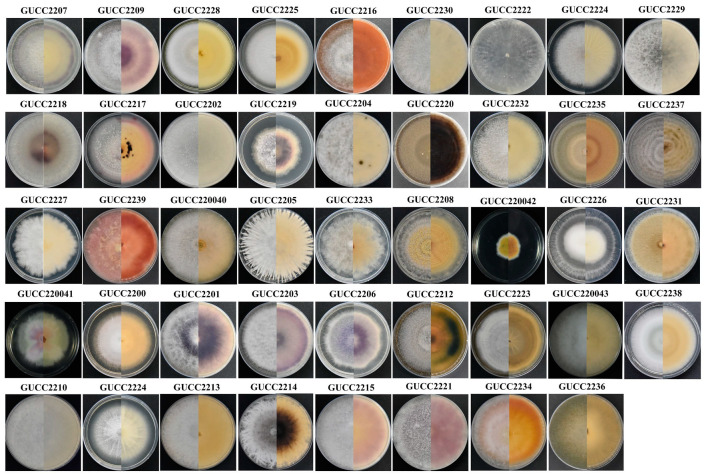
Forty-four fungal strains with different morphological characteristics.

Of the 200 examined cysts of *G. rostochiensis*, 80% were colonized by one to five or more different species of fungi, and 139 culturable strains were obtained by isolation and purification (Table 1). The pure cultures were initially grouped based on their morphological criteria. The morphological characteristics selected for the observation were based on colonial color, mycelial shape, and growth rate. Among these strains, the colors were found to be: white, yellow, orange, red, gray pink, gray, purple, gray to brown, and brown to black. Various mycelial forms, such as compact, cottony, and airy, were observed. After seven days of cultivation, the mycelial growth rate of one strain (GUCC220042) was much lower than all other strains, only reaching 2 cm (diameter). Finally, forty-four fungal strains with different morphological characteristics were selected (Figure 2). In total, 139 fungal strains were identified based on ITS sequence analyses and morphological observations. Forty-four isolates with different morphological characteristics were identified through multigene analysis of the combined internal transcribed spacer (ITS), 28S large subunit rDNA (LSU), and beta-tubulin (TUB). All sequences of the isolates were analyzed by NCBI-BLAST, representing 23 different genera.

### 3.2. Diversity of Colonizing Fungi

#### 3.2.1. Dominant Taxa

**Figure 3:**
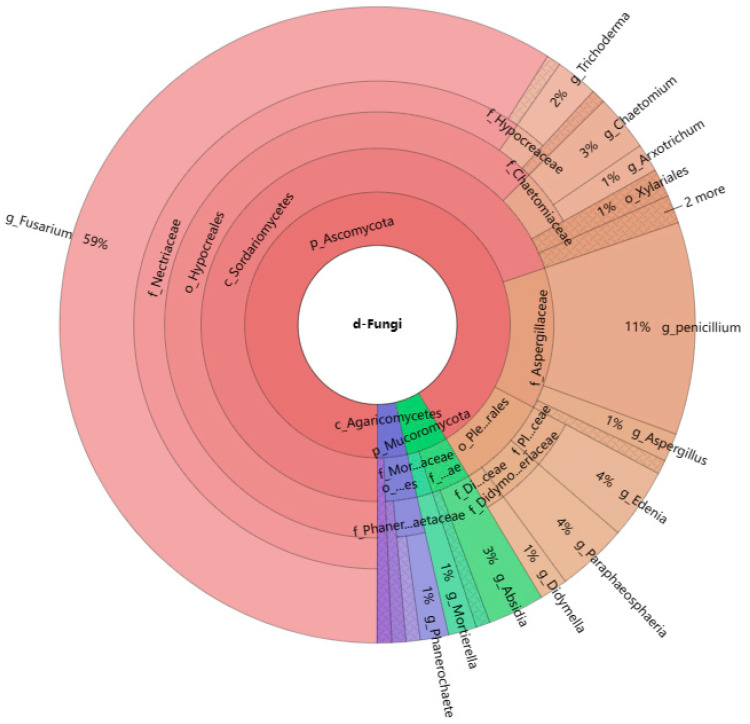
Species composition of the colonizing fungi from *G. rostochiensis***.** The letters before each scientific name at the different taxonomic levels represent the corresponding taxonomic levels: P—phylum, C—class, O—order, F—family, and G—genus.

In all the isolates of the colonizing fungi, the Camargo index (1/S) at the order, family, and genus levels were 0.091, 0.059, and 0.043, respectively. Therefore, the dominant order was Hypocreales (62.6%); the dominant families were Nectriaceae (59.7%) and Aspergillaceae (12.9%); of the fungi identified, most were strains of *Fusarium* (58.9%) or *Penicillium* (10.8%). *Fusarium* was associated with 82 cysts, and *Penicillium* colonized 11% of the cysts. *Absidia* (2.9%), *Arxotrichum* (1.4%), *Aspergillus* (1.4%), *Chaetomium* (2.9%), *Clonostachys* (0.7%), *Coriolopsis* (0.7%), *Crinipellis* (0.7%), *Didymella* (1.4%), *Edenia* (3.6%), *Gongronella* (0.7%)*, Mortierella* (1.4%), *Nigrospora* (0.7%), *Paecilomyces* (0.7%), *Paraphaeosphaeria* (3.6%), *Peroneutypa* (0.7%), *Pestalotiopsis* (0.7%), *Phaeophlebiopsis* (0.7%), *Phanerochaete* (1.4%), *Trichoderma* (2.2%), *Volutella* (0.7%) and *Xylaria* (0.7%) were infrequently associated with the cysts. At the same time, the proportion of fungi in each genus is clear (Figure 3). All the isolated fungi emerged from anywhere on the cyst surface.

#### 3.2.2. Lifestyle Diversity

**Figure 4:**
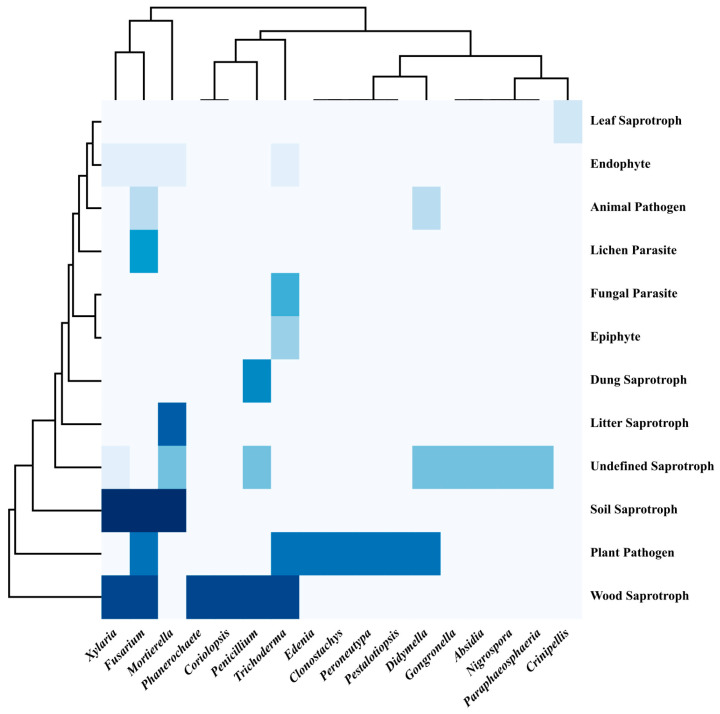
Heatmap of the functional annotations of colonizing fungi. Different blue shades indicate different fungal lifestyles.

Twenty-three genera of colonizing fungi were analyzed for functional annotation in the FUNGuild database. No information was obtained for six genera—*Arxotrichum*, *Aspergillus*, *Chaetomium*, *Paecilomyces*, *Phaeophlebiopsis,* and *Volutella*. There were twelve different lifestyles represented by the remaining genera. Functional annotations of other genera are described in Figure 4. Plant pathogens and saprotrophs (wood, soil, and undefined saprotrophs) dominated the fungal communities, followed by endophytic fungi (4/23). Four genera, namely *Fusarium*, *Mortierella*, *Trichoderma*, and *Xylaria*, were found to have four or more lifestyles. *Didymella* and *Fusarium* are animal pathogens and can infect animals. *Trichoderma* are the only fungal parasites.

### 3.3. Parasitic Potential of the Fungal Isolates towards Nematode Cysts In Vitro

**Figure 5:**
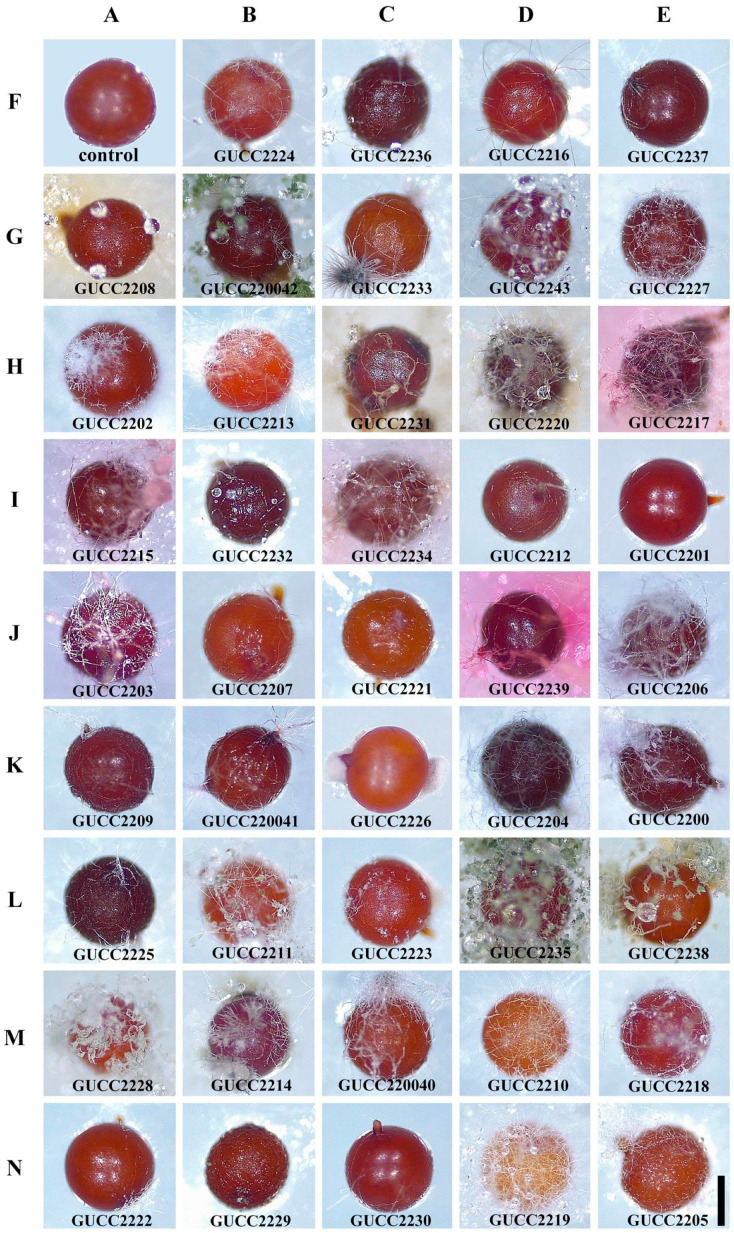
The parasitic potential of 44 fungal isolates on cysts of *G. rostochiensis*. AF: control. BF–EN: fungal strains that colonized the cysts. Scale bar: AF–EN = 1 mm.

Forty-four fungal strains of varied morphology had different colonization rates on cysts. Most strains had a high colonization rate on cysts of *G. rostochiensis* (100%); the lowest was 16.7% (Table 2). Among the 44 fungi, the colonization rate of 40 fungal isolates to cysts was greater than 50%, especially since there were 27 strains with a colonization rate of 100% and only four fungi (61.4%) with a colonization rate less than 50% to cysts (Table 3). We found that strains belonging to *Arxotrichum* (GUCC2216, GUCC2237), *Aspergillus* (GUCC2208, GUCC220042), *Chaetomium* (GUCC2233, GUCC220043), *Clonostachys* (GUCC2227), *Coriolopsis* (GUCC2202), *Crinipellis* (GUCC2213), *Didymella* (GUCC2231, GUCC2220), *Edenia* (GUCC2217), *Gongronella* (GUCC2224)*, Nigrospora* (GUCC2204), *Paecilomyces* (GUCC2200), *Pestalotiopsis* (GUCC220040), and *Trichoderma* (GUCC2229, GUCC2230) had higher colonization rates. *Mortierella* (GUCC2226) and *Phanerochaete* (GUCC2218, GUCC2222) had low isolation and colonization rates. However, there were also *Absidia* (GUCC2236), *Peroneutypa* (GUCC2214), *Phaeophlebiopsis* (GUCC2210), and *Volutella* (GUCC2219) which had a moderate ability to colonize cysts. These genera have significant biocontrol potential and deserve further study of their other biocontrol effects. Fungal strains colonizing the cysts (Figure 5). The first cyst was a control, and the other cysts showed the colonizing phenomena of different fungal strains. Other differences included colony position, degree of colonization, and fungal growth on the cyst’s surface after colonization. However, in general, the most common sites of infection by the colonizing fungi were the natural orifices of the cysts, such as the mouth or anus.

## 4. Discussion

A total of 139 fungal strains were found on the cysts of *G. rostochiensis* from Weining County, Guizhou Province, China. The fungal isolates were assigned to three phyla, 11 orders, 17 families, and 23 genera. These fungi belonged to the genera *Fusarium*, *Penicillium*, *Absidia*, *Arxotrichum*, *Aspergillus*, *Chaetomium*, *Clonostachys*, *Coriolopsis*, *Crinipellis*, *Didymella*, *Edenia*, *Gongronella*, *Mortierella*, *Nigrospora*, *Paecilomyces*, *Paraphaeosphaeria*, *Peroneutypa*, *Pestalotiopsis*, *Phaeophlebiopsis*, *Phanerochaete*, *Trichoderma*, *Volutella,* and *Xylaria*. Forty-four strains with different morphological characteristics were selected from 139 fungi, which all had a certain colonization rate to cysts in vitro. In 44 strains, 27 demonstrated a colonization rate of 100% on the cysts of *G. rostochiensis.* Colonized fungi were isolated from *G. rostochiensis* for the first time in China, and the taxonomic diversity of the fungi from *G. rostochiensis* were preliminarily clarified.

In previous studies, there have been many reports on colonizing fungi associated with cyst nematodes, most of them concerning *Heterodera* spp., with only a few on *Globodera* spp. In Siwi’s study, out of 123 fungal isolates obtained from PCN cysts and PCN-infested soils in Indonesia, 12 isolates showed egg- and cyst-parasitic abilities of over approximately 50%, which were identified as *Gliocladium virens*, *F. oxysporum*, *F. lateritium*, *P. tritinum*, *P. oxalicum*, and *Taralomyces* spp. [55]. The fungi isolated in this study were mostly opportunistic fungi, a class of fungi that specifically or facultatively colonize the cysts of plant-parasitic nematodes, including a large number of soil-dwelling fungi [56]. *Fusarium*, *Paecilomyces,* and *Mortierella*, the most commonly isolated genera, were also isolated in this study [57]. Combining the results of previous studies, we found that the dominant genera on the cysts of PCNs were *Fusarium* and *Penicillium*. *Fusarium* was the most abundant genus. Although the isolation rate of *Fusarium* on cysts is very high, whether *Fusarium* is pathogenic to potatoes and whether it is suitable as a common plant-pathogenic fungus for biocontrol fungi remains to be further studied.

In our fungal isolates, we found pathogens of plant diseases from genera such as *Pestalotiopsis* [58,59], *Volutella* [60,61], *Didymella* [62], *Xylaria* [63], and *Crinipellis* [64]; the genus *Coriolopsis* which is associated with trees and one of the lignicolous fungi [65]; and *Paraphaeosphaeria* [66], *Peroneutypa* [67], and *Phaeophlebiopsis* [68], which are genera associated with the epiphytic fungi of plants. The fungi of the genus *Absidia* are usually isolated from soil, constituting the pathogens of many human diseases [69,70,71,72]. *Gongronella* is capable of hydrolyzing polymeric chitosan to produce oligomeric chitosan [73]. White-rot fungus *Phanerochaete sordida* of the genus *Phanerochaete* has a high biodegradation efficiency in the degradation process of microorganisms [74]. Previous studies have revealed a few reports of the genera *Edenia* and *Arxotrichum*. The fungi of the genera *Aspergillus* [75], *Fusarium* [76], *Penicillium* [77], *Trichoderma* [78], *Paecilomyces* [79], *Chaetomium* [80], *Mortierella* [81], *Nigrospora* [82], and *Clonostachys* [83] act as biocontrol agents for the management of plant-parasitic nematodes. Overall, we found a higher diversity of fungi in the cysts of *G. rostochiensis* in Weining County valuable to study.

The percentages of cysts, eggs, and females of cyst nematodes colonized by fungi in agricultural soil ranged from 10 to 90%, with about 50% being the most common species [84,85]. In this study, we obtained 40 isolates showing cyst-colonizing abilities of over 50%. Currently, two possible routes for the biological management of plant-parasitic nematodes have been proposed. One is to mass produce an effective nematode-destroying fungus in the laboratory and then apply it to soils [86]. The other is to enhance the natural nematophagous fungal populations in soils by altering their surrounding conditions. However, the commercial success of these approaches has been limited; nevertheless, there are encouraging reports concerning reducing nematode populations by adding certain kinds of amendments, such as chitin and green manure crops, to soils [87,88].

Although not considered as traditional biological control, another promising approach by which nematophagous fungi, as well as other soil fungi, can be used for developing new means to control animal- and plant-parasitic nematodes is to use antagonists as a source for isolating new compounds with nematicidal activity [89]. Our study focuses on the diversity and parasitic potential of colonizing fungi isolated from *G. rostochiensis* in order to better understand their ecology. For the development and stability of nematode biocontrol agents, it is critical to analyze the biocontrol potential of these colonizing fungi and the soil ecology of the colonized microorganisms.

## Figures and Tables

**Table 1:** The genera composition of *G. rostochiensis* cysts colonized by fungi from Weining County.

Genus	Culturable Strains	
	Number	Percentage (%)
*Absidia*	4	2.88
*Arxotrichum*	2	1.44
*Aspergillus*	2	1.44
*Chaetomium*	4	2.88
*Clonostachys*	1	0.72
*Coriolopsis*	1	0.72
*Crinipellis*	1	0.72
*Didymella*	2	1.44
*Edenia*	5	3.60
*Fusarium*	82	58.99
*Gongronella*	1	0.72
*Mortierella*	2	1.44
*Nigrospora*	1	0.72
*Paecilomyces*	1	0.72
*Paraphaeosphaeria*	5	3.60
*Penicillium*	15	10.79
*Peroneutypa*	1	0.72
*Pestalotiopsis*	1	0.72
*Phaeophlebiopsis*	1	0.72
*Phanerochaete*	2	1.44
*Trichoderma*	3	2.16
*Volutella*	1	0.72
*Xylaria*	1	0.72

**Table 2:** Colonization rate of 44 fungal strains on cysts of *G. rostochiensis*.

Strain Number	Relative Colonization Rate (%) ± Standard Error	Strain Number	Relative Colonization Rate (%) ± Standard Error
GUCC2225	16.67 ± 5.77	GUCC2208	100.00 ± 0
GUCC2226	20.00 ± 0	GUCC2209	100.00 ± 0
GUCC2221	33.33 ± 5.77	GUCC2211	100.00 ± 0
GUCC2222	33.33 ± 5.77	GUCC2212	100.00 ± 0
GUCC2201	66.67 ± 5.77	GUCC2213	100.00 ± 0
GUCC2218	69.23 ± 5.77	GUCC2215	100.00 ± 0
GUCC2205	78.57 ± 5.77	GUCC2216	100.00 ± 0
GUCC2207	78.57 ± 5.77	GUCC2217	100.00 ± 0
GUCC2239	80.00 ± 10	GUCC2224	100.00 ± 0
GUCC2219	80.00 ± 0	GUCC2227	100.00 ± 0
GUCC2236	85.71 ± 5.77	GUCC2228	100.00 ± 0
GUCC2214	86.67 ± 5.77	GUCC2229	100.00 ± 0
GUCC2220	87.50 ± 5.77	GUCC2230	100.00 ± 0
GUCC220041	91.67 ± 0	GUCC2231	100.00 ± 0
GUCC2223	93.33 ± 5.77	GUCC2232	100.00 ± 0
GUCC220043	93.33 ± 5.77	GUCC2233	100.00 ± 0
GUCC2210	93.33 ± 5.77	GUCC2234	100.00 ± 0
GUCC2200	100.00 ± 0	GUCC2235	100.00 ± 0
GUCC2202	100.00 ± 0	GUCC2237	100.00 ± 0
GUCC2203	100.00 ± 0	GUCC2238	100.00 ± 0
GUCC2204	100.00 ± 0	GUCC220040	100.00 ± 0
GUCC2206	100.00 ± 0	GUCC220042	100.00 ± 0

**Table 3:** The proportion of fungal strains with their colonization rate.

Relative Colonization Rate (R)	Percentage
<50%	9.09%
50% ≤ R < 80%	9.09%
80% ≤ R < 100%	20.45%
=100%	61.36%

## Data Availability

Not applicable.

## References

[1] Zaheer K., Akhtar M.H. (2016). Potato production, usage, and nutrition—A review. Crit. Rev. Food Sci..

[2] Zhang H., Fen X.U., Yu W.U., Hu H.H., Dai X.F. (2017). Progress of potato staple food research and industry development in China. J. Integr. Agr..

[3] Barma N.C.D., Hossain A., Hakim M., Mottaleb K.A., Alam M., Reza M., Rohman M. (2019). Progress and challenges of wheat production in the era of climate change: A Bangladesh perspective. Wheat Production in Changing Environments.

[4] Price J.A., Coyne D., Blok V.C., Jones J.T. (2021). Potato cyst nematodes *Globodera rostochiensis* and *G. pallida*. Mol. Plant Pathol..

[5] Turner S. (1998). The origins, global distribution and biology of potato cyst nematodes (*Globodera rostochiensis* (Woll.) and *Globodera pallida* Stone). Potato Cyst Nematodes, Biology, Distribution and Control.

[6] Evans K., Stone A.R. (1977). A review of the distribution and biology of the potato cyst-nematodes *Globodera rostochiensis* and *G. pallida*. Pans.

[7] Nicol J.M., Turner S.J., Coyne D.L., Den Nijs L., Hockland S., Maafi Z.T., Jones J.T., Gheysen G., Fenoll C. (2011). Current nematode threats to world agriculture. Genomics and Molecular Genetics of Plant-Nematode Interactions.

[8] Pineda O., Bonierbale M.W., Plaisted R.L., Brodie B.B., Tanksley S.D. (1993). Identification of RFLP markers linked to the H1 gene conferring resistance to the potato cyst nematode *Globodera rostochiensis*. Genome.

[9] Devrajan K., Prabhu S., Seenivasan N., Sudha A., Ramakrishnan S., Anita B. (2011). Occurrence of native microbial antagonists against potato cyst nematodes in the Nilgiri Hills of Tamil Nadu. Potato J..

[10] Krishna Prasad K.S., Singh D.B. (1986). Note on the parasitic nematodes associated with potato in Karnataka State, India. Inter. Nematol. Network Newsletter.

[11] Ramana K.V., Mohandas C. (1988). Occurrence of potato cyst nematode *Globodera pallida* (Stone, 1973) in Kerala. Indian J. Nematol..

[12] Nagachandrabose S. (2017). Combined application of *Pseudomonas fluorescens* and *Purpureocillium lilacinum* liquid formulations to manage *Globodera* spp. on potato. Crop Prot..

[13] Jiang R., Huan P., Li Y. (2022). First record of the golden potato nematode *Globodera rostochiensis* in Yunnan and Sichuan provinces of China. J. Integr. Agr..

[14] Peng D.L., Liu H., Peng H., Jiang R., Li Y.Q., Wang X., Ge J.J., Zhao S.Q., Feng X.D., Feng M.Y. (2022). First detection of the potato cyst nematode (*Globodera rostochiensis*) in a major potato production region of China. Plant Dis..

[15] Gray N.F. (1983). Ecology of nematophagous fungi: Distribution and habitat. Ann. Appl. Biol..

[16] Yu Q., Coosemans J. (1998). Fungi associated with cysts of *Globodera rostochiensis*, *G. pallida*, and *Heterodera schachtii*; and egg masses and females of *Meloidogyne hapla* in Belgium. Phytoprotection.

[17] Kerry B.R. (1990). An assessment of progress toward microbial control of plant-parasitic nematodes. J. Nematol..

[18] Fresenius G. (1854). Beitrage zur Mykologie. J. Cell Sci..

[19] Kerry B.R. (1988). Fungal parasites of cyst nematodes. Agric. Ecosyst. Environ..

[20] Nordbring-Hertz B., Jansson H.B., Tunlid A. (2006). Nematophagous fungi. Encycl. Life Sci..

[21] Song J., Li S., Xu Y., Wei W., Yao Q., Pan F. (2016). Diversity of parasitic fungi from soybean cyst nematode associated with long-term continuous cropping of soybean in black soil. Acta Agric. Scand. Sect. B Soil Plant Sci..

[22] Liu X.Z., Chen S.Y. (2011). Screening isolates of *Hirsutella* species for biocontrol of *Heterodera glycines*. Biocontrol. Sci. Technol..

[23] Ma R., Liu X.Z., Jian H., Li S.D. (2005). Detection of *Hirsutella* spp. And *Pasteuria* sp. parasitizing second-stage juveniles of *Heterodera glycinesin* soybean fields in China. Biol. Control..

[24] Rajeswari S., Sivakumar C.V. (1999). Nematophagous fungi associated with the potato cyst nematodes, *Globodera* spp. in the Nilgiris, Tamil Nadu. Biol. Control..

[25] Chen S.Y., Liu X.Z. (2005). Control of soybean cyst nematode by the fungi *Hirsutella rhossiliensis* and *Hirsutella minnesotensis* in greenhouse studies. Biol. Control..

[26] Borneman J., Becker O. (2007). Identifying microorganisms involved in specific pathogen suppression in soil. Annu. Rev. Phytopathol..

[27] Alabouvette C., Olivain C., Migheli Q., Steinberg C. (2009). Microbiological control of soil-borne phytopathogenic fungi with special emphasis on wilt-inducing *Fusarium oxysporum*. New Phytol..

[28] Diaz-Silveira M.F., Herrera J.O. (1998). An overview of nematological problems in Cuba. Nematropica.

[29] Mittal N., Saxena G., Mukerji K.G. (1995). Integrated control of root-knot disease in three crop plants using chitin and Paecilomyces lilacinus. Crop Prot..

[30] Franco J., Jatala P., Bocangel M. (1981). Efficiency of *Paecilomyces lilacinus* as a biological agent of *Globodera pallida*. J. Nematol..

[31] Westphal A., Becker J.O. (2001). Components of soil suppressiveness against *Heterodera schachtii*. Soil Biol. Biochem..

[32] Holland R.J., Williams K.L., Khan A. (1999). Infection of *Meloidogyne javanica* by *Paecilomyces lilacinus*. Nematology.

[33] Siddiqui Z.A., Mahmood I. (1996). Biological control of plant parasitic nematodes by fungi: A review. Bioresour. Technol..

[34] Dunn M.T., Sayre R.M., Carrell A., Wergin W.P. (1984). Colonization of nematode eggs by *Paecilomyces lilacinus* (Thom) Samson as observed with scanning electron-microscopy. Scan. Electron Micros..

[35] Dackman C. (1990). Fungal parasites of the potato cyst nematode *Globodera rostochiensis*: Isolation and reinfection. J. Nematol..

[36] Crump D.H., Flynn C.A. (1995). Isolation and screening of fungi for the biological control of potato cyst nematodes. Nematologica.

[37] Oro V., Boskovic J., Milenkovic S., Tosi S. (2012). Taxonomic diversity of fungi associated with some PCN populations from Serbia. Pestic. Phytomed..

[38] Abbasi K., Zafari D., Wick R. (2017). Evaluation of chitinase enzyme in fungal isolates obtained from golden potato cyst nematode (*Globodera rostochiensis*). Zemdirbyste.

[39] Benttoumi N., Colagiero M., Sellami S., Boureghda H., Keddad A., Ciancio A. (2020). Diversity of nematode microbial antagonists from Algeria shows occurrence of nematotoxic *Trichoderma* spp.. Plants.

[40] Zhang X., Zhang H., Jiang Z., Bai Q., Wu S., Wang Y., Li C., Zeng X., Gan X., Xie X. (2021). A new strain of *Volutella citrinella* with nematode predation and nematicidal activity, isolated from the cysts of potato cyst nematodes in China. BMC Microbiol..

[41] Vieira Dos Santos M.C., Horta J., Moura L., Pires D., Conceicao I., Abrantes I., Costa S.R. (2019). An integrative approach for the selection of *Pochonia chlamydosporia* isolates for biocontrol of potato cyst and root knot nematodes. Phytopathol. Mediterr.

[42] Nagachandrabose S. (2020). Management of potato cyst nematodes using liquid bioformulations of *Pseudomonas fluorescens*, *Purpureocillium lilacinum* and *Trichoderma viride*. Potato Res..

[43] Duan S.M., Huang X.F., Hu J.W., Xia P.H., Wang Y., Liu Y.Y. (2013). Study on Speciation of Phosphorus in Rhizosphere Sediments from Caohai Wetland in Guizhou Province. Res. Environ. Sci..

[44] Zhu Z.J., Chen J.A., Zeng Y. (2014). Paleotemperature variations at Lake Caohai, southwestern China, during the past 500 years: Evidence from combined δ18O analysis of cellulose and carbonates. Sci. China Earth Sci..

[45] Mo M.H. (1995). Advance on Ecological Status of Nematophagous Fungi. Guizhou Agric. Sci..

[46] Southey J.F. (1974). Methods for detection of potato cyst nematodes. Bull. OEPP.

[47] Been T.H., Schomaker C.H. (2000). Development and evaluation of sampling methods for fields with infestation foci of potato cyst nematodes (*Globodera rostochiensis* and *G. pallida*). Phytopathology.

[48] Reid A., Pickup J. (2005). Molecular characterization of a morphologically unusual potato cyst nematode. Bull. OEPP.

[49] Nurjanah Trisyono Y.A., Indarti S., Hartono S. (2016). Identification, distribution and genetic diversity of the golden potato cyst nematode (*Globodera rostochiensis*) in Java Indonesia. AIP Conf. Proc..

[50] Baunacke W. (1922). Investigations on biology and control of Beet nematodes, *Heterodera schachtii* Schmidt. Arb. Aus Der Biol. Reichsanst..

[51] Hallman J., Viaene N. (2013). PM 7/119 (1) Nematode extraction. Bull. OEPP.

[52] Hall T.A. (1999). BioEdit: A user-friendly biological sequence alignment editor and analysis program for Windows 95/98/NT. Nucleic Acids Symp. Ser..

[53] Camargo J.A. (1992). Can dominance influence stability in competitive interactions?. Oikos.

[54] Nguyen N.H., Song Z., Bates S.T., Branco S., Tedersoo L., Menke J., Schilling J.S., Kennedy P.G. (2016). FUNGuild: An open annotation tool for parsing fungal community datasets by ecological guild. Fungal Ecol..

[55] Indarti S., Widianto D., Kim Y.H., Mulyadi M., Suryanti S. (2010). Survey of egg-and cyst-parasitic fungi of potato cyst nematode in Indonesia. Plant Pathol. J..

[56] Liu X.Z., Zhang K.Q., Li T.F. (2004). Biological Control of Plant Parasitic Nematodes.

[57] Zhang Y., Li G.H., Zhang K.Q. (2011). A review on the research of nematophagous fungal species. Mycosystema.

[58] Qi M., Xie C.X., Chen Q.W., Yu Z.D. (2021). *Pestalotiopsis trachicarpicola*, a novel pathogen causes twig blight of *Pinus bungeana* (Pinaceae: Pinoideae) in China. Anton. Leeuw. Int. J. G..

[59] Nozawa S., Seto Y., Watanabe K. (2019). First report of leaf blight caused by *Pestalotiopsis chamaeropis* and *Neopestalotiopsis* sp. in Japanese andromeda. J. Gen. Plant Pathol..

[60] Šafránková I. (2007). Volutella leaf blight and stem canker on Japanese pachysandra in the Czech Republic. Plant Protect. Sci..

[61] Shi F., Hsiang T. (2014). *Pseudonectria buxi* causing leaf and stem blight on *Buxus* in Canada. Eur. J. Plant Pathol..

[62] Cui Y., Peng A., Song X., Cheng B., Ling J., Chen X. (2021). First report of chick peach (*Prunus persica* L.) leaf spot disease caused by *Didymella glomerata* in China. Plant Pathol..

[63] Kousalya S., Kamalakannan A., Chowdappa A., Gopalakrishnan C., Rajamani K., Venkatesh A. (2019). First report of *Xylaria* sp. causing tuber rot on glory lily in India. New Dis. Rep..

[64] Cui B.K. (2008). A species of lignicolous Fungi New to China—*Coriolopsis glabro-rigens*. J. For. Res..

[65] Marco J.L.D., Valadares-Inglis M.C., Felix C.R. (2003). Production of hydrolytic enzymes by *Trichoderma* isolates with antagonistic activity against *Crinipellis perniciosa*, the causal agent of witches’ broom of cocoa. Braz. Microbiol..

[66] Phookamsak R., Hyde K.D., Jeewon R., Bhat D.J., Jones E.G., Maharachchikumbura S.S., Xu J. (2019). Fungal diversity notes 929–1035: Taxonomic and phylogenetic contributions on genus and species of fungi. Fungal Divers..

[67] De Errasti A., Novas M.V., Carmarán C.C. (2014). Plant-fungal association in trees: Insights into changes in ecological strategies of *Peroneutypa scoparia* (*Diatrypaceae*). Flora.

[68] Santos T.F.B., dos Santos Carvalho C., de Almeida M.A., Delforno T.P., Duarte I.C.S. (2020). Endophytic fungi isolated from Brazilian medicinal plants as potential producers of antioxidants and their relations with anti-inflammatory activity. 3 Biotech.

[69] Cordeiro L., Lee H.B., Nguyen T.T.T., Gurgel L.M.S., de Azevedo A.L.C.M. (2021). *Absidia bonitoensis* (Mucorales, Mucoromycota), a new species isolated from the soil of an upland Atlantic forest in Northeastern Brazil. Nova Hedwigia..

[70] Albert Q., Leleyter L., Lemoine M., Heutte N., Rioult J.P., Sage L., Baraud F., Garon D. (2018). Comparison of tolerance and biosorption of three trace metals (Cd, Cu, Pb) by the soil fungus *Absidia cylindrospora*. Chemosphere.

[71] Sebastian K.K., Alzayer H., Abraham E., Roche D., Reddan D., Lappin D. (2021). Cutaneous *Absidia corymbifera* in a Lupus Nephritis Patient. Cureus.

[72] Cloughley R., Kelehan J., Corbett-Feeney G., Murray M., Callaghan J., Regan P., Cormican M. (2002). Soft tissue infection with *Absidia corymbifera* in a patient with idiopathic aplastic anemia. J. Clin. Microbiol..

[73] Nwe N., Furuike T., Osaka I., Fujimori H., Kawasaki H., Arakawa R., Tokura S., Stevens W.F., Kurozumi S., Takamori Y. (2011). Laboratory scale production of 13c labeled chitosan by fungi *Absidia coerulea* and *Gongronella butleri* grown in solid substrate and submerged fermentation. Carbohydr. Polym..

[74] Wang J., Suzuki T., Mori T., Yin R., Dohra H., Kawagishi H., Hirai H. (2021). Transcriptomics analysis reveals the high biodegradation efficiency of white-rot fungus *Phanerochaete sordida* YK-624 on native lignin. J. Biosci. Bioeng..

[75] Xalxo P.C., Karkun D., Poddar A.N. (2013). Rhizospheric fungal associations of root knot nematode infested cucurbits: In vitro assessment of their nematicidal potential. Res. J. Microbiol..

[76] Devi G., Bora L. (2018). Effect of some biocontrol agents against root-knot nematode (*Meloidogyne incognita* race2). Int. J. Environ. Agric. Biotechnol..

[77] Sikandar A., Zhang M., Wang Y., Zhu X., Liu X., Fan H., Duan Y. (2020). In vitro evaluation of *Penicillium chrysogenum* Snef1216 against *Meloidogyne incognita* (root-knot nematode). Sci. Rep..

[78] Gogoi D., Mahanta B. (2013). Comparative efficacy of *Glomus fasciculatum*, *Trichoderma harzianum*, carbofuran and carbendazim in management of *Meloidogyne incognita* and *Rhizoctonia solani* disease complex on French bean. Ann. Plant Prot. Sci..

[79] Baheti B.L., Dodwadiya M., Bhati S.S. (2017). Eco-friendly management of maize cyst nematode, *Heterodera zeae* on sweet corn (*Zea mays* L.. saccharata). J. Entomol. Zool. Stud..

[80] Nitao J.K., Meyer S.L., Oliver J.E., Schmidt W.F., Chitwood D.J. (2002). Isolation of flavipin, a fungus compound antagonistic to plant-parasitic nematodes. Nematology.

[81] Gortari C., Cazau C., Hours R. (2007). Hongos nematófagos de huevos de Toxocara canis en un paseo público de La Plata, Argentina. Rev. Iberoam. Micol..

[82] Amin N. (2015). Nematicidal activity of root exudates of sengon plant inoculated with endophytic fungi *Nigrospora* sp. to control of root-knot nematode *Meloidogyne* spp.. J. Chem. Pharm. Res..

[83] Zhao M.L., Huang J.S., Mo M.H., Zhang K.Q. (2005). A potential virulence factor involved in fungal pathogenicity: Serine-like protease activity of nematophagous fungus *Clonostachys rosea*. Fungal Divers..

[84] Tribe H.T. (1980). Extent of disease in populations of *Heterodera*, with special reference to *H. schachtii*. Ann. Appl. Biol..

[85] Clovis C.J., Nolan R.A. (1983). Fungi associated with cysts, eggs and juveniles of the golden nematode (*Globodera rostochiensis*) in Newfoundland. Nematologica.

[86] Coosemans J. (1991). Methods for introducing *Verticillium chlamydosporium* into soil. IOBC/WPRS Bull..

[87] Spiegel Y., Chet I., Cohn E. (1987). Use of chitin for controlling plant-parasitic nematodes. II. Mode of action. Plant Soil.

[88] Schlang J.W., Stendel J.W., Muller J. Influence of resistant green manure crops on the population dynamics of *Heterodera schachtii* and its fungal egg parasites. Proceedings of the European Society of Nematologists 19th International Nematology Symposium.

[89] Anke H., Stadler M., Mayer A., Sterner O. (1995). Secondary metabolites with nematicidal and antimicrobial activity from nematophagous fungi and Ascomycetes. Can. J. Bot..

